# Mental Health Symptoms in Oral Contraceptive Users During Short-Term Hormone Withdrawal

**DOI:** 10.1001/jamanetworkopen.2023.35957

**Published:** 2023-09-27

**Authors:** Isabel A. Noachtar, Vibe G. Frokjaer, Belinda Pletzer

**Affiliations:** 1 Department of Psychology and Centre for Cognitive Neuroscience, University of Salzburg, Salzburg, Austria; 2 Neurobiology Research Unit, Copenhagen University Hospital Rigshospitalet, Copenhagen, Denmark; 3 Psychiatric Center Copenhagen, Mental Health Services Capital Region of Denmark, Copenhagen, Denmark; 4 Department of Clinical Medicine, University of Copenhagen, Copenhagen, Denmark

## Abstract

**Importance:**

Hormonal contraception has been linked to mood symptoms and the ability to recognize emotions after short periods of treatment, whereas the mental health of users of long-term hormonal contraceptives has had limited investigation.

**Objective:**

To evaluate whether short-term hormonal withdrawal, which users of combined oral contraceptives (COCs) undergo once a month (pill pause), was associated with altered mood and emotional recognition in long-term users of COCs.

**Design, Setting, and Participants:**

This case-control study included a community sample of individuals assigned female sex at birth who identified as women and used COC for 6 months or longer. The control group included women with natural menstrual cycles who otherwise fulfilled the same inclusion criteria. The study was conducted between April 2021 and June 2022 in Salzburg, Austria.

**Exposure:**

COC users and women with natural menstrual cycles were tested twice within a month, once during their active pill phase or luteal phase and once during their pill pause or menses.

**Main Outcomes and Measures:**

Negative affect, anxiety, and mental health problems were assessed during each session. The percentage increase in mental health symptoms was calculated during the pill pause compared with that during the active intake phase in COC users. How this change compared with mood fluctuations along the menstrual cycle in women with natural menstrual cycles was assessed.

**Results:**

A total of 181 women aged 18 to 35 years (mean [SD] age, 22.7 [3.5] years) were included in the analysis (61 women with androgenic COC use, 59 with antiandrogenic COC use, 60 women with a menstrual cycle not taking COCs). COC users showed a 12.67% increase in negative affect (95% CI, 6.94%-18.39%), 7.42% increase in anxiety (95% CI, 3.43%-11.40%), and 23.61% increase in mental health symptoms (95% CI, 16.49%-30.73%; *P* < .001) during the pill pause compared with the active intake phase. The effect size of this change did not differ depending on progestin type (negative affect: *F*_1,117_ = 0.30, *P* = .59; state anxiety: *F*_1,117 _= 2.15, *P* = .15; mental health: *F*_1,117 _= .16, *P* = .69) or ethinylestradiol dose (negative affect: *F*_1,57_ = .99, *P* = .32; state anxiety: *F*_1,57_ = 2.30, *P* = .13; mental health: *F*_1,57_ = .14, *P* = .71) was comparable with mood changes along the menstrual cycle in women with natural cycles (negative affect: *F*_2,175_ = 0.13, *P* = .87; state anxiety: *F*_2,175_ = 0.14, *P* = .32; mental health: *F*_2,175_ = 0.65, *P* = .52). Mood worsening during the pill pause was more pronounced in women with higher baseline depression scores (negative affect increase of 17.95% [95% CI, 7.80%-28.10%] in COC users with higher trait depression [BDI >8]). Emotion recognition performance did not differ between active pill phase and pill pause.

**Conclusions and Relevance:**

In this case-control study of long-term COC users, withdrawal from contraceptive steroids during the pill pause was associated with adverse mental health symptoms similar to those experienced by women during menses with withdrawal from endogenous steroids. These results question the use of the pill pause from a mental health perspective. Long-term COC users may benefit more from the mood-stabilizing effects of COCs in cases of continuous intake.

## Key Points

**Question:**

Is short-term hormone withdrawal, or pill pause, associated with changes in mental health symptoms in long-term users of combined oral contraceptives (COCs) in a manner depending on progestin type, estrogen dose, and baseline mental health symptoms?

**Findings:**

This case-control study including 180 women found significant mood deterioration during the pill pause. The effect size was comparable with mood changes across the menstrual cycle and more pronounced in COC users with higher baseline depression.

**Meaning:**

Similar mood worsening is seen after withdrawal from exogenous hormones in oral contraceptives as from endogenous hormones during menses; therefore, whether a pill pause is advantageous from a mental health perspective is questionable.

## Teaser

This case-control study evaluates if short-term hormone withdrawal of oral contraceptives (pill pause) is associated with changes in mental health symptoms in long-term users of combined oral contraceptives depending on progestin type, estrogen dose, and baseline mental health symptoms.

## Introduction

According to the United Nations Sexual and Reproductive Health Agency, women worldwide are in need of reliable and safe contraceptive options. Approximately 407 million women use hormonal contraceptives worldwide.^1^ The mental health implications of hormonal contraception are heavily debated since a Danish registry study^2^ including over 1 million women estimated that during the first few months of combined oral contraceptive (COC) use, the risk for a first diagnosis of depression or antidepressant use increases 1.8-fold, and suicides or suicide attempts increase approximately 2-fold.^3^ Increased depression rates among COC users were more pronounced in adolescent women, as also replicated in a study by Zettermark et al.^4^ However, this association between depression and COC use was not replicated in other studies of similar scale,^5,6^ and results from nationally representative samples from Finland^7,8^ and the US^9,10^ even suggest mental health benefits of COC use, as evidenced by reduced rates of panic disorder,^9^ depression,^7,8,10^ and suicide, especially in adolescent COC users.^10^

Given this diversity in cross-sectional findings, it is unclear whether mental health is influenced by COC use per se or related to associated factors such as changes in lifestyle, sexual activity, relationship status, or socioeconomic status. To predict causality between COC use and mood, placebo-controlled, randomized clinical trials (RCTs) using different COC formulations were conducted. Most of these trials observed significantly lower mental well-being during the first few months of COC intake^11,12,13,14,15^ (except for a study by Scheuringer et al^16^), although only a few studies reported an increase in clinical depression scores.^15,17,18^

Two factors may contribute to those seemingly contradictory results between non-RCTs and RCTs. First, the specific formulation of COCs may play a role regarding the likelihood of adverse mood effects. For example, the current literature hints at fewer adverse effects on mood with COCs containing less androgenic progestins.^18,19,20^ Likewise, a lower ethinylestradiol dose was found to be associated with improved mood.^21^

Second, individual characteristics may predispose some women to adverse mood symptoms, while protecting others. Accordingly, the different study designs may capture different populations of women. Placebo-controlled trials usually report a mood worsening within the first 6 months of COC treatment.^11,13,15^ Population-based studies, on the other hand, usually report improved mental health in long-term users of COCs (>6 months of COC use).^6,8,10^ These discrepancies arise because adverse effects on mood are one of the most common reasons for discontinuation.^19^ Accordingly, the group of long-term users are usually women who tolerate COCs well, known as the *survivor effect*. In line with this idea, population-based studies report decreased depression rates with longer duration of COC use.^2,6,7^ Unfortunately, only a few studies have explored what differentiates women who experience adverse effects during the first 6 months of treatment from women who experience beneficial effects. COC-associated adverse mood symptoms were especially found in women who were younger when initializing COC use or had a history of mental health issues or induced abortion.^13,14,22,23^ However, there is accumulating evidence that some women may display heightened hormonal sensitivity, resulting in mental health problems during phases of hormonal change (study review performed by Schweizer-Schubert^24^). For example, postpartum depression is more likely in women who also experience premenstrual mood symptoms^25^ or mental health problems during COC use.^26^

Interestingly, mental health problems associated with endogenous hormonal change appear most commonly during phases of hormonal withdrawal, like the premenstrual or postpartum period or menopause.^27^ Also, withdrawal from hormone replacement therapy results in negative mood symptoms.^28^ However, neither RCTs nor cohort studies have the necessary temporal resolution to identify whether mental health symptoms occur in response to COC application or during COC withdrawal. This is of particular relevance because the majority of COC formulations are designed to artificially mimic a menstrual cycle. Thus, over a period of 28 days, hormone-containing COC pills are taken for 21 to 24 days, followed by a so-called *pill pause*, ie, 4 to 7 days during which no pills are taken or during which the COC pills are replaced by placebo pills. During this pill-free interval, women experience exogenous hormonal withdrawal, which is accompanied by a vaginal bleeding similar to menses. Thus, even though mental health benefits in long-term COC users have been attributed to mood stabilization due to the constant hormonal milieu elicited by the daily intake of the same contraceptive formulation, this stable hormonal milieu is disrupted once a month to elicit a withdrawal bleeding. Although much attention has been paid to mental health symptoms in COC users, it has always been assumed that they occur during active intake. Accordingly, almost no research, to our knowledge, has been conducted into the mental health implications of the pill pause.

In the current study, we explored whether the pill pause is accompanied by changes in mental health symptoms in a well-powered sample of long-term COC users and whether these changes are moderated by (1) progestin type (androgenic vs antiandrogenic), (2) estrogen dose, and (3) previous mental health symptoms. By including a control group of women with natural menstrual cycles, we assessed how these changes compared with changes in mental health symptoms along the endogenous menstrual cycle.

## Methods

### Study Design

This case-control study received approval from the ethical committee of the University of Salzburg and conformed to the Code of Ethics of the World Medical Association (Declaration of Helsinki). Written informed consent to participate in the study was given by all participants. This study followed the Strengthening the Reporting of Observational Studies in Epidemiology (STROBE) reporting guidelines.^29^

### Participants

We recruited a community sample of long-term COC users (≥6 months), who were right-handed, fluent in German, aged between 18 and 35 years, did not take medication besides the COC, and had no psychiatric, endocrinologic, or neurologic illnesses. Information on participant race and ethnicity was not obtained because the study protocol did not provide approval access to this information. All participants were assigned female sex at birth and identified as women. Users of COCs with the progestins levonorgestrel, desogestrel, gestodene, etonogestrel, or norelgestromin were classified as androgenic COC users (COC-A).^30^ Users of COCs containing dienogest, drospirenone, chlormadinone acetate, cyproterone acetate, or nomegestrol acetate were classified as antiandrogenic COC users (COC-AA).^30^ No restrictions were based on the estrogen dosage. Control participants, ie, women with natural menstrual cycles (NCs), were selected from a previous study^31^ and had to fulfill the same inclusion criteria as COC users with the exception of not having taken COCs for the past 6 months. In addition, they had to show a regular menstrual cycle of 21 to 35 days with a maximum of 7 days of variability between individual cycles.^32^

### Procedure

The study was carried out between April 2021 and June 2022 in Salzburg, Austria. Participants were alerted to the study via flyers and social media posts. They could sign up via an online administration tool and had to pass a series of filter questions to be registered as eligible participants, who then received a standardized email including more detailed information on the study. If they agreed to participate, a first study date was scheduled. COC users were each tested twice, once during the second or third week of their active intake phase (intake days 9-21) and once during their pill pause. Half of the participants completed their first session during the active pill phase; the other half completed their first session during the pill pause. To ensure an adequate washout period for the synthetic steroids, pill pause sessions were scheduled on the fourth to seventh day of the pill pause. We aimed to schedule follow-up visits within a pill cycle; although for 10 participants, follow-up visits had to be rescheduled due to the COVID-19 pandemic. For control participants, we used a sample of women with NCs from a previous study on menstrual cycle–associated changes in mood, including 3 cycle phases.^31^ For the current study, we selected sessions scheduled during menses as corresponding to the low-hormone pill pause and sessions scheduled during the midluteal cycle phase as corresponding to the high-hormone active pill phase. Half of the participants completed the luteal session before the menses session; the other half completed the menses session before the luteal session. Midluteal sessions had previously been confirmed by backward counting of cycle days and elevated progesterone levels.^31^ During each appointment, participants completed a health screening, 3 cognitive tasks (verbal fluency, navigation, mental rotation, published in a study by Noachtar and Pletzer),^33^ an emotion recognition task, mood questionnaires, and provided 3 saliva samples over the course of 90 minutes. Trait measures for mental health over the past month before study participation, an IQ estimate (Raven advanced progressive matrices^34^), and demographics were assessed at the first appointment. The current study focused on the mood questionnaires.

### Mood Questionnaires

To assess mood, state and trait measures were performed (eMethods in Supplement 1). Trait measures included the Premenstrual Symptom Screening Tool (PSST),^35^ Beck Depression Inventory (BDI-II),^36^ and Beck Anxiety Inventory (BAI)^37^ and were only administered during the first session irrespective of cycle/pill phase because they assess symptoms covering the last 4 weeks before study participation. State measures were performed during each session and included the Positive and Negative Affect Schedule (PANAS),^38^ an emoji scale assessing positive and negative affect (A.M. Beltz, PhD, University of Michigan, written communication, October 26, 2019), the Daily Rating of Severity of Problems (DRSP),^39^ and the state version of the State-Trait Anxiety Inventory (STAI).^40^

### Statistical Analysis

We planned our group size to ultimately allow for the comparison of 3 groups (COC-A, COC-AA, NC) across 2 time points. Power analyses were carried out using G*Power software, version 3.1.9.7 (Axel Buchner).^41^ Because we were interested in 6 dependent variables (3 state mood scales, 3 emotion recognition measures), uncorrected α error probability was set to 0.008. Assuming moderate effect sizes and a correlation of 0.5 between repeated measurements, a power of 80% requires a total sample size of 180 participants.

The hypotheses and analysis plan were preregistered on September 26, 2022, on the platform AsPredicted.^42^ R software, version 4.1.0 (R Core Team) was used to compute the statistical analysis. Linear mixed effects models (LMEs) were performed using the lme function of the nlme package.^43^ In each model, we included age, session, and IQ as covariates and participant number (PNr) as a random factor. In a first step, the analyses were restricted to COC users. To examine if self-reported mood (E/PANAS composite score, STAI score, DRSP score) and emotion recognition (accuracy, speed, count) differed between active intake and pill pause, the factor phase (active/pause) was included in all models. Within the same models, we also assessed how this difference was moderated by the androgenicity of the progestin (model 1: mood ~ phase × androgenicity + session + age + IQ + 1│PNr). Beyond the scope of the preregistration, we explored whether baseline mood values presented a significant confound because participants with better mood during the active intake phase had more potential for mood worsening. Accordingly, model 1 was rerun by including mood scores during the active intake phase as a covariate (model 1a: mood ~ phase × androgenicity + session + age + IQ + baseline mood +1│PNr). To assess whether the phase difference was modulated by the ethinylestradiol dose, ethinylestradiol dose was added as an additional predictor in a second model (model 2: mood ~ phase × ethinylestradiol dose + session + age + IQ + 1│PNr). Because ethinylestradiol dose only showed sufficient variation among androgenic COC users, this analysis had to be restricted to androgenic COC users. To examine if self-reported mood changes from active intake phase to pill pause were moderated by baseline depression or anxiety, BDI and BAI scores, respectively, were included as additional predictors in a third model (model 2: mood ~ phase × BDI/BAI + session + age + IQ + 1│PNr). Finally, to assess how mood changes during the pill pause compared with differences along the menstrual cycle in those not taking COCs, the analyses were extended and the first model was rerun, including a 3-way group factor (model 1b: mood ~ phase × group + session + age + IQ + 1│PNr). In all models, the analysis of variance function of the statistical package, followed by pairwise Tukey tests, was conducted using the glht function of the multcomp package,^44^ which was used to determine the significance of main effects. In accordance with the a priori power analyses, 2-sided *P* values were considered significant at a Bonferroni corrected level of *P* < .008. To support null effects, Bayes factors were calculated using the lmBF function of the BayesFactor package.^45^ Specifically, we calculated the Bayes factor for a model without the factor/interaction term relative to the model including the factor/interaction term using 10 000 iterations for Monte Carlo sampling. Accordingly, Bayes factors (BF_01_) are reported including the percentage variation among the 10 000 iterations. For quantification purposes, we calculated the percentage increase in negative affect, anxiety, and mental health symptoms during the pill pause.

## Results

### Demographics

As outlined in the supplementary flowchart (eFigure in Supplement 1), a total of 181 women aged 18 to 35 years (mean [SD] age, 22.7 [3.5] years) were included in the analysis (61 women with androgenic COC use, 59 with antiandrogenic COC use, 60 women with a menstrual cycle not taking COCs). Of the 155 COC users included in the study, 35 were lost to follow-up. The control sample consisted of 60 NC participants with a mean (SD) cycle length of 28.7 (2.3) days. Thus, as required by the power analysis, 180 participants were included in the statistical analysis. Table 1 provides an overview of the progestins and estrogen dosage contained in currently used COCs, as well as information on previous COC use and basic demographics. The majority of participants was heterosexual, nulliparous, and nonsmoking; in addition, they had passed general qualification for university entrance and were not currently employed (Table 1). To account for minor differences in age and IQ (compare Table 1), these variables were used as covariates in subsequent analyses.

**Table 1.** Demographic Data^a^

Variable	Androgenic COC (n = 61)	Antiandrogenic COC (n = 59)	Nonusers (n = 60)	*P* value for group comparison
Current COC use, No. of participants (% sample) [hormone]				
Progestin type	48 (78.7) [levonorgestrel]	32 (54.2) [dienogest]	NA	NA
	9 (14.8) [gestoden]	6 (10.2) [drospirenone]		
	4 (6.6) [desogestrel]	16 (27.1) [CMA]		
		3 (5.1) [CPA]		
		2 (3.4) [NGA]		
EE dose^b^	0 (0) [EE, 0.035 mg]	3 (5.1) [EE, 0.035 mg]		
	26 (43.3) [EE, 0.03 mg]	50 (84.7) [EE, 0.03 mg]		
	32 (53.3) [EE, 0.02 mg]	2 (3.4) [EE, 0.02 mg]		
	2 (3.3) [EE, 0.015 mg]			
COC pause/cycle day for menses, d				
Mean (SD)	5.79 (0.97)	5.48 (1.06)	4.12 (1.18)	NA
Median (IQR)	6.00 (2.00)	5.00 (1.00)	4.00 (2.00)	
COC intake/cycle day for luteal phase, d				
Mean (SD)	13.64 (2.03)	13.38 (2.15)	22.69 (2.49)	
Median (IQR)	14.00 (3.00)	13 (3.00)	22.50 (3.25)	
Previous COC use, No. of participants (% sample)				
No prior COC use	56 (92)	48 (81)	27 (45)	NA
Prior androgenic COC use	0 (0)	6 (10)	13 (22)	
Prior antiandrogenic COC use	5 (8)	5 (9)	14 (23)	
Total hormonal contraception duration, mo				
Mean (SD)	52.34 (32.63)	63.34 (44.65)	24.43 (37.69)	NA
Median (IQR)	47.00 (49.00)	53.00 (48.00)	6.00 (37.50)	
Demographics				
Age, median (IQR), y	23 (5)	21 (3.5)	21 (3)	.002
IQ, median (IQR)	106 (19)	106 (12)	106 (19)	.02
Education, No. of participants (% sample)				
University qualification	55 (90)	56 (95)	59 (98)	.15
Employment status, No. of participants (% sample)				
No employment	40 (66)	43 (73)	36 (60)	.29
Part time	13 (21)	9 (15)	19 (32)	
Full time	8 (13)	7 (12)	5 (8)	
Sexual orientation, No. of participants (% sample)				
Heterosexual	56 (92)	54 (92)	50 (83)	.39
Bisexual	5 (8)	5 (8)	9 (15)	
Homosexual	0 (0)	0 (0)	1 (2)	
Relationship status, No. of participants (% sample)				
In relationship	42 (69)	34 (58)	34 (57)	.33
Parity, No. of participants (% sample)				
Nulliparous	61 (100)	59 (100)	59 (98)	.67
Smoking status, No. of participants (% sample)				
Nonsmoker	55 (90)	53 (90)	57 (95)	.56

Abbreviations: CMA, chlormadinone acetate; COC, combined oral contraceptive; CPA, cyproterone acetate; EE, ethinylestradiol; NA, not applicable; NGA, nomegestrol acetate.

^a^ Age and IQ were compared between groups using Wilcoxon signed ranks tests. All other demographic variables were compared using χ^2^ tests. *P* values were false discovery rate corrected across demographic variables.

^b^ A total of 4 antiandrogenic COCs did not contain EE, rather, they contained other estrogens.

### Group Differences in Trait Measures of Mental Health

The mean (SD) trait measures of mental health did not differ significantly between the 2 COC groups and NC women (BDI score: COC-A, 7.1 [5.4]; COC-AA, 10.2 [7.1]; NC, 8.5 [7.2]; *P* = .03; BAI score: COC-A, 7.6 [5.9]; COC-AA, 8.8 [6.9]; NC, 8.0 [5.5]; *P* > .99; PSST score: COC-A, 9.7 [5.2]; COC-AA, 9.7 [6.3]; NC, 12.1 [5.6]; *P* = .03) (Table 2).

**Table 2.** Self-Reported Trait and State Mood During the Combined Oral Contraceptive (COC) Phases or Menstrual Cycle Presented as Mean (SD)^a^

Group	BDI	BAI	PSST	Positive affect		Negative affect		STAI		DRSP	
				Active/ luteal	Pause/menses	Active/ luteal	Pause/menses	Active/ luteal	Pause/menses	Active/ luteal	Pause/menses
COC-A	7.1 (5.4)	7.6 (5.9)	9.7 (5.2)	3.5 (0.7)	3.3 (0.7)	1.7 (0.4)	1.9 (0.6)	1.7 (0.3)	1.8 (0.4)	1.9 (0.6)	2.3 (0.8)
COC-AA	10.3 (7.1)	8.9 (6.9)	9.7 (6.4)	3.4 (0.8)	3.3 (0.7)	1.8 (0.6)	2.0 (0.6)	1.8 (0.4)	1.8 (0.4)	2.1 (0.7)	2.5 (0.8)
NC	8.5 (7.2)	8.0 (5.5)	12.1 (5.6)	3.3 (0.8)	3.0 (0.8)	1.8 (0.6)	2.0 (0.7)	1.8 (0.4)	1.9 (0.5)	2.1 (0.9)	2.6 (0.9)

Abbreviations: BAI, Beck Anxiety Inventory; BDI, Beck Depression Inventory; COC-A, androgenic COC users; COC-AA, antiandrogenic COC users; DRSP, Daily Rating of Severity of Problems; NC, natural cycle; PSST, Premenstrual Symptom Screening Tool; STAI, State-Trait Anxiety Inventory.

^a^ Positive and negative affect scores were assessed on a 5-point Likert-scale, STAI scores on a 4-point Likert scale, and DRSP scores on a 6-point Likert-scale. The maximum BDI/BAI score is 63; the maximum PSST score is 54.

### Mood Changes During the Pill Pause and Their Moderation by Pill Type

**Figure 1.**
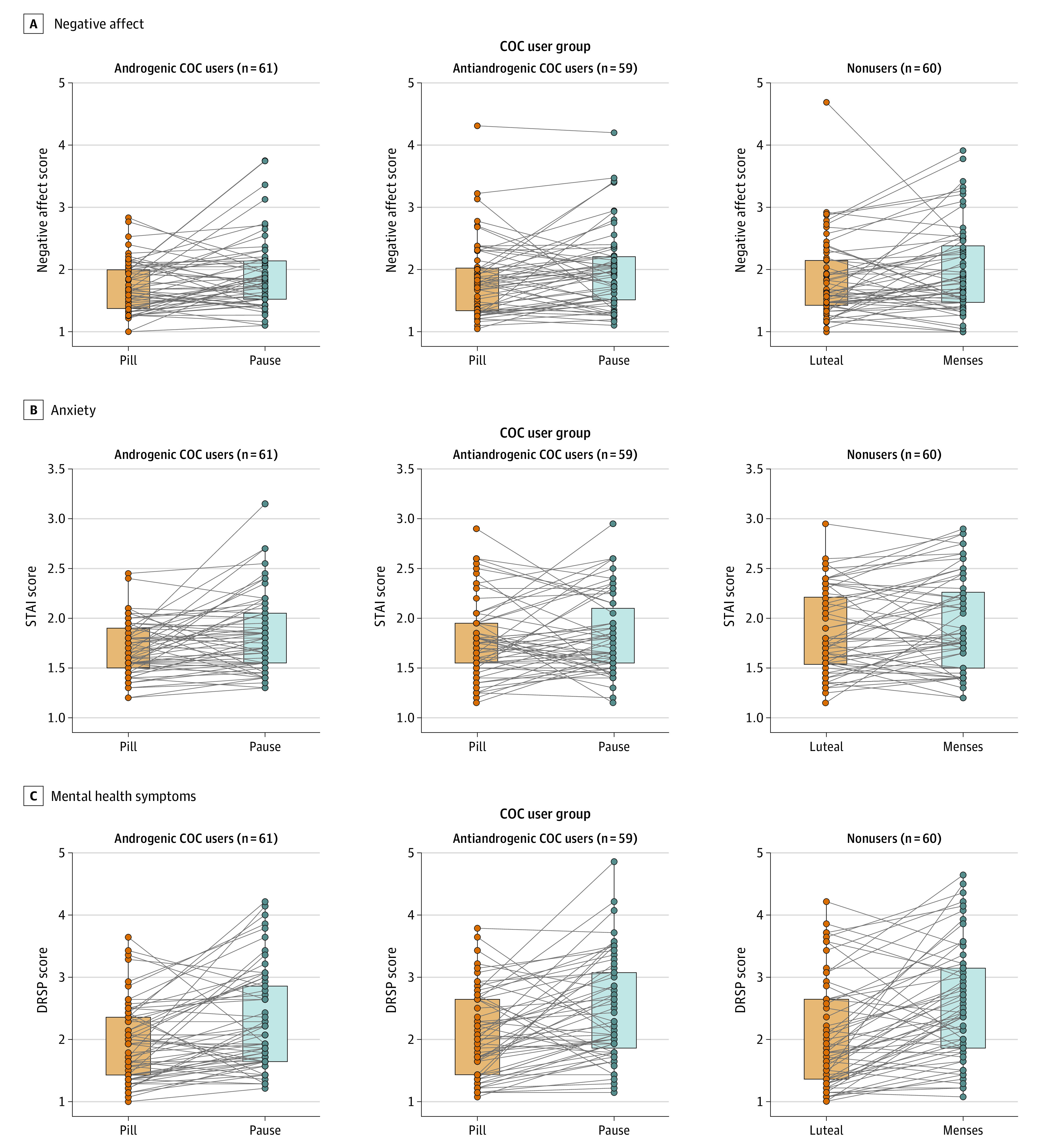
Self-Reported Mood During Active Pill Intake vs Pill Pause in Long-Term Combined Oral Contraceptive (COC) Users Data are presented separately for androgenic and antiandrogenic COC users and in comparison with mood changes along the menstrual cycle in women with natural menstrual cycles. Negative affect (A), anxiety (B), and mental health symptoms (C) increased significantly during the pill pause compared with active intake irrespective of OC type. Effect size was comparable with mood changes along the menstrual cycle.

No difference in emotion recognition was found between the active and pill pause conditions (eAppendix in Supplement 1). During the pill pause, COC users showed a 12.67% increase in negative affect (95% CI, 6.94%-18.39%; *P* = .009), 7.42% increase in anxiety (95% CI, 3.43%-11.40%; *P* = .003), and 23.61% increase in mental health symptoms (95% CI, 16.49%-30.73%; *P* < .001) (Figure 1). Positive affect was nonsignificantly reduced by 1.76% (95% CI, −6.15% to 2.64%; *P* = .03). The change during the pill pause did not differ between androgenic and antiandrogenic COC users (negative affect: *F*_1,117_ = 0.30, *P* = .59; state anxiety: *F*_1,117_ = 2.15, *P* = .15; mental health: *F*_1,117_ = 0.16, *P* = .69) and was not associated with ethinylestradiol dose in androgenic COC users (negative affect: *F*_1,57_ = 0.99, *P* = .32; state anxiety: *F*_1,57_ = 2.30, *P* = .13; mental health: *F*_1,57_ = 0.14, *P* = .71). Including baseline mood scores as potential confounders in the models did not change these results.

### Moderation of Mood Changes During Pill Pause by Trait Depression and Anxiety

**Figure 2.**
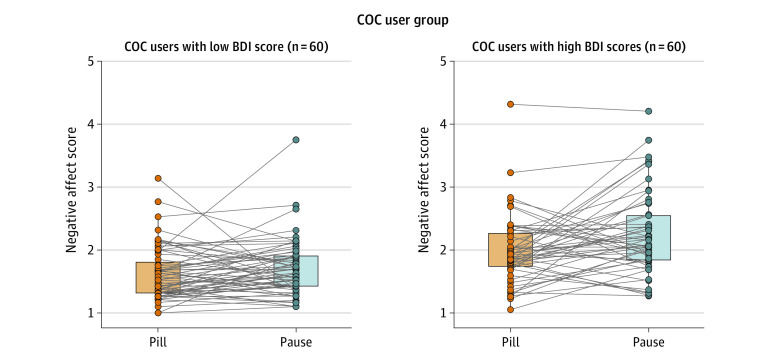
Moderation of Mood Worsening During Pill Pause by Trait Depression There was an associated increase in negative affect during the pill pause in women with high trait depression (Beck Depression Inventory [BDI] >8) scores compared with women with low trait depression scores (BDI ≤8).

A significant interaction between trait depression (BDI) and phase was found for negative affect (*F*_1,117_ = 7.14; *P* = .008). Although negative affect increased during the pill pause by 8.48% (95% CI, 2.04%-14.93%) in COC users with lower trait depression (BDI score ≤8), the increase amounted to 17.95% (95% CI, 7.80%-28.10%) in COC users with higher trait depression (BDI score >8) (Figure 2). Trait anxiety (BAI) did not moderate changes in state anxiety during the pill pause (*F*_1,117_ = 0.39; *P* = .53).

### Comparison of Mood Changes During Pill Pause to Menstrual Cycle-Related Changes

When NC women were included, the group did not emerge as a significant moderator of self-reported mood changes across phases (negative affect: *F*_2,175_ = 0.13, *P* = .87; mean [SD] BF_01_ = 16.55% [2.19%]; state anxeity: *F*_2,175_ = 0.14, *P* = .32; mean [SD] BF_01_ = 6.55% [2.48%]; mental health: *F*_2,175_ = 0.65, *P* = .52; mean [SD] BF_01_ = 9.95% [1.48%]). Comparable with changes during the pill pause in COC users, negative affect increased by 12.93% (95% CI, 3.37%-22.49%), anxiety by 6.83% (95% CI, 1.61%-12.06%), and mental health symptoms by 33.80% (95% CI, = 21.56%-46.05%) from the high hormone luteal phase to the low hormone menses in NC women (Figure 1).

## Discussion

The aim of the current study was to assess whether short-term withdrawal from synthetic hormones during the pill pause was associated with mood and emotion recognition changes and to assess relevant moderators of these changes. Our data suggest a significant increase in negative affect, anxiety, and mental health symptoms during the pill pause in long-term COC users, which was (1) irrespective of COC type, (2) moderated by baseline depression scores, and (3) comparable with mood changes observed along the NC.

This result is in line with the assumption of a mood-stabilizing effect of COCs in long-term users^46,47^ and questions the usefulness of frequent, ie, monthly, pill pauses from a mental health perspective. It is possible that this mood worsening is related to previously observed changes in brain structure and resting state connectivity during the pill pause.^48,49,50^ Adverse mood symptoms during the pill pause may also explain why results on the effectiveness of COCs as a treatment for premenstrual dysphoric disorder are mixed.^51^

Mood changes were comparable between androgenic and antiandrogenic COC users and irrespective of ethinylestradiol dose, suggesting that the type of progestin does not play a role in mental health symptoms associated with hormone withdrawal. It cannot be determined at present whether the mood worsening is associated with withdrawal from estrogens or progestins. Nevertheless, based on our data, the progestin actions on androgen receptors do not seem to play a significant role in that respect.

Importantly, mood worsening during the pill pause was not only comparable between pill types but also with the mood worsening observed in NC women during their menses, suggesting similar effects of both synthetic and endogenous hormonal withdrawal. Given that any type of hormone withdrawal appears to elicit similar symptom strength, it is also possible that the increase in mental health symptoms was not a direct result of hormone withdrawal but was associated with the physical discomfort during withdrawal bleeding.

Finally, mood changes during the pill pause were more pronounced in women with higher trait depression scores as assessed with the BDI. Although it has been reported that women with a history of depression are more susceptible to negative mood symptoms during short-term COC use,^13,14,52^ to the best of our knowledge, this was the first study demonstrating a similar effect in long-term COC users.

### Limitations

This study has some limitations. Our results may not generalize to women who experience mental health problems during the first few months of COC use because only long-term COC users who tolerate their COC well were included in the present study. Unfortunately, we have not collected data on previous adverse effects during COC use in either group. In addition, only broad categories of COC type were compared because the study was underpowered for further subgroup analyses. Accordingly, follow-up studies are necessary to determine the specific effects of hormone withdrawal for each contraceptive formulation.

## Conclusions

In this case-control study, repeated monthly withdrawal from contraceptive steroids during the pill pause was associated with similar mental health symptoms as those of endogenous hormonal withdrawal during menses, irrespective of the contraceptive formulation. These results question the usefulness of pill pauses from a mental health perspective, and it should be explored whether long-term COC users benefit more from the mood-stabilizing effects of COCs in cases of continuous intake.

## Notes

Supplement 1. **eMethods.** Mental Health Questionnaires **eFigure.** Participant Flow Chart **eAppendix.** Emotion Recognition Task
eReferences

Supplement 2.
Data Sharing Statement
