# Supplementary material for: Mental Health Symptoms in Oral Contraceptive Users During Short-Term Hormone Withdrawal
Source: JAMA Netw Open. 2023 Sep 27;6(9):e2335957. doi: 10.1001/jamanetworkopen.2023.35957 (PMC10534273; doi:10.1001/jamanetworkopen.2023.35957)
Supplement: Supplement 1. — eMethods. Mental Health Questionnaires eFigure. Participant Flow Chart eAppendix. Emotion Recognition Task eReferences [file jamanetwopen-e2335957-s001.pdf]

Supplementary Online Content

Noachtar IA, Frokjaer VG, Pletzer B. Mental health symptoms in oral contraceptive users during short-term hormone withdrawal. *JAMA Netw Open*. 2023;6(9):e2335957. doi:10.1001/jamanetworkopen.2023.35957

eMethods. Mental Health Questionnaires
eFigure. Participant Flow Chart
eAppendix. Emotion Recognition Task
eReferences

This supplementary material has been provided by the authors to give readers additional information about their work.

© 2023 Noachtar IA et al. *JAMA Network Open.*

eMethods. Mental health questionnaires

The *Premenstrual Symptom Screening Tool* (PSST)[1] consists of 20-items aimed at assessing the severity of premenstrual symptoms; 14 items examine symptoms like depression, anxiety, mood lability, irritability, concentration difficulties, sleep problems and a variety of monthly reoccurring physical symptoms on a 4-point Likert scale. The PSST score was calculated by averaging the 14 items and Cronbach's Alpha for the current sample was 0.85.

The German version of the *Becks Depression Inventory* (BDI)[2] was conducted during the first session to assess depression scores. The 21 items cover the diagnostic criteria for depression according to the DSM-V.[3] Participants may select one of four statements reflecting varying degrees of symptom strength for each item (0-3). In this study, the score was determined by summing up the raw score of the 21 item responses.

The *Becks Anxiety Inventory* (BAI)[4] was used to determine trait anxiety. On a 4-point Likert scale, participants were instructed to rate the severity of psychological and somatic anxiety symptoms during the past weeks. The score was determined by summing up the raw score of the 21 item responses.

The Daily Rating of Severity of Problems (DRSP)[5] is a rating scale to monitor the intensity and impact of psychological and physical symptoms on a 6-point Likert Scale. The DRSP was adapted to include 20 items and 14 items were averaged to determine the DRSP score during each session. Cronbach's Alpha for the current sample was 0.87.

The *Positive and Negative Affect Schedule* (PANAS)[6] assessed the participant's current affect. The PANAS has been widely used in menstrual cycle studies and consists of 20 items with 10 positive and 10 negative affective adjectives. In accordance with their current mood, participants rate the adjectives on a 5-point Likert scale. In the current sample, Cronbach's alpha for positive affect was 0.88 and Cronbach's alpha for negative affect was 0.82.

An Emoji scale assessing positive and negative affect (A.M. Beltz, PhD, University of Michigan, written communication, October 26, 2019) was included to aid the younger generation of participants. The scale consists of 16-items (six positive and ten negative emojis) and participants had to indicate how much each emoji corresponds with their current mood on a 5-point Likert scale. In this sample, Cronbach's alpha for positive affect was 0.93 and Cronbach's alpha for negative affect was 0.92. Positive and negative affect scores of the PANAS and emoji scale were highly correlated (positive affect: $r = 0.67$, $p < .0001$; negative affect: $r = 0.78$, $p < 0.001$). A composite score for negative affect was calculated by averaging the negative affect scores of the PANAS and emoji scale and a composite score for positive affect was calculated by averaging the positive affect scores of the PANAS and emoji scale.

© 2023 Noachtar IA et al. *JAMA Network Open.*

The *State Trait Anxiety Inventory* (STAI)[7] was used to assess participants' current level of anxiety. The STAI consists of 20 adjectives related to the psychological or physical symptoms of anxiety or nervousness or their opposite, i.e., relaxation, which participants rated on a 4-point Likert scale. The Cronbach's alpha was 0.86.

eFigure. Participant Flow Chart

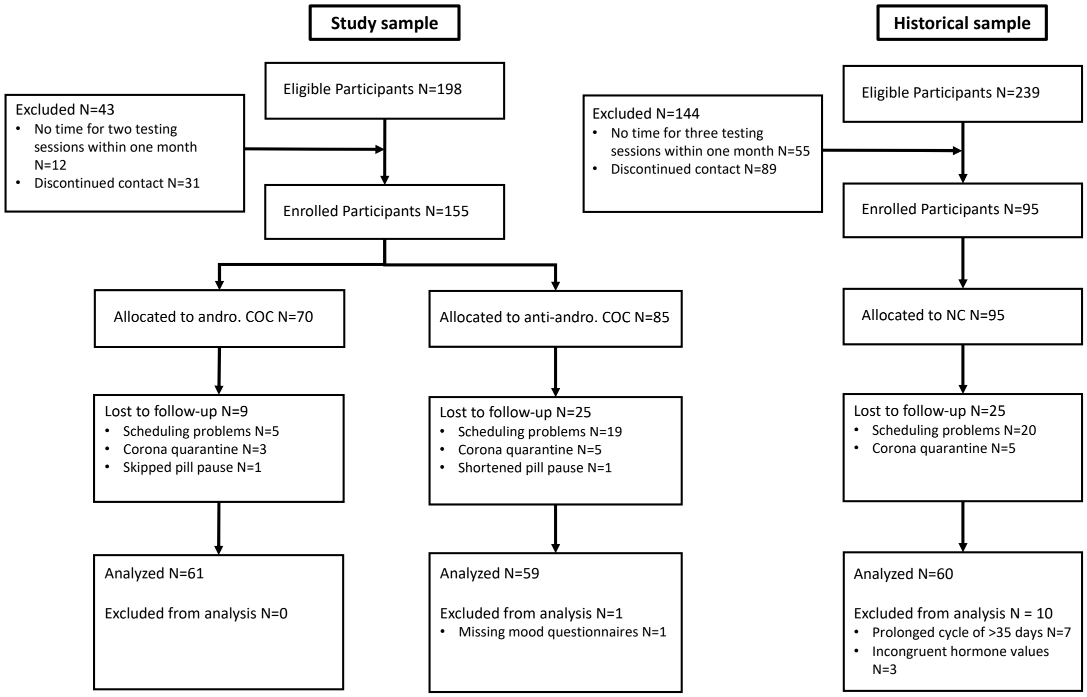

Andro. COC = androgenic combined oral contraceptives, anti-andro. COC = anti-androgenic combined oral contraceptives, NC = natural cycle

eAppendix. Emotion Recognition Task

During each session, participants were presented with 60 human faces selected from the FACES database[8] displaying one of six basic emotional expressions (happiness, fear, anger, sadness, disgust, neutral) using OpenSesame 3.2.8 (stimulus duration: 4000 milliseconds (ms); inter-stimulus interval: 495 ms). Participants were instructed to indicate the correct emotion by key press as fast as possible. We quantified the speed of responses (reaction times in ms), accuracy of responses, as well as frequency with which each emotional expression was selected, irrespective of the emotional expression displayed.

Irrespective of the emotion displayed, no emotion recognition measure differed between active pill intake and pill pause, irrespective of androgenicity (all $F < 1.68$, all $p > 0.2$). Bayes factors provide strong evidence that emotion recognition performance did not differ between groups (all $BF_{01} > 106.67 \pm 3.63\%$) and phases (all $BF_{01} > 10.69 \pm 0.88\%$).

© 2023 Noachtar IA et al. *JAMA Network Open.*

eReferences

1. Steiner M, Macdougall M, Brown E. The premenstrual symptoms screening tool (PSST) for clinicians. *Arch Womens Ment Health*. 2003;6:203-209. doi:10.1007/s00737-003-0018-4
2. Hautzinger M, Keller F, Kühner C. *Beck Depressions-Inventar (BDI-II)*. Harcourt Test Services; 2006.
3. American Psychiatric Association D, Association AP. *Diagnostic and Statistical Manual of Mental Disorders: DSM-5*. Vol 5. American psychiatric association Washington, DC; 2013.
4. Margraf J, Beck AT, Ehlers A. *Beck-Angst-Inventar: BAI; Manual*. Harcourt Test Services; 2007.
5. Endicott J, Nee J, Harrison W. Daily Record of Severity of Problems (DRSP): reliability and validity. *Arch Womens Ment Health*. 2006;9:41-49. doi:10.1007/s00737-005-0103-y
6. Krohne HW, Egloff B, Kohlmann CW, Tausch A. Untersuchungen mit einer deutschen version der "positive and negative affect schedule"(PANAS). *Diagnostica*. 1996;42:139-156.
7. Laux L, Glanzmann P, Schaffner P, Spielberger CD. *Das State-Trait-Angstinventar (Testmappe Mit Handanweisung, Fragebogen STAI-G Form X 1 Und Fragebogen STAI-G Form X 2)*. Beltz; 1981. Accessed August 20, 2023. https://diabetes-psychologie.de/downloads/Beschreibung_STAI.pdf
8. Ebner NC, Riediger M, Lindenberger U. FACES: A database of facial expressions in young, middle-aged, and older women and men (publicly available datasets). *Max Planck Society*. Published online 2018. doi:10.3758/BRM.42.1.351

© 2023 Noachtar IA et al. *JAMA Network Open.*
