# Supplementary material for: Mental Health Symptoms in Oral Contraceptive Users During Short-Term Hormone Withdrawal
Source: JAMA Netw Open. 2023 Sep 27;6(9):e2335957. doi: 10.1001/jamanetworkopen.2023.35957 (PMC10534273; doi:10.1001/jamanetworkopen.2023.35957)
Supplement: Supplement 2. — Data Sharing Statement [file jamanetwopen-e2335957-s002.pdf]

Data Sharing Statement

Noachtar. Mental Health Symptoms in Oral Contraceptive Users During Short-Term Hormone Withdrawal. *JAMA Netw Open*. Published September 27, 2023.
doi:10.1001/jamanetworkopen.2023.35957

Data
Data available: Yes
Data types: Deidentified participant data, Data dictionary
How to access data: https://osf.io/9g6y7/
When available: With publication

Supporting Documents
Document types: Statistical/analytic code
How to access documents: https://osf.io/9g6y7/
When available: With publication

Additional Information
Who can access the data: anyone
Types of analyses: for any purpose
Mechanisms of data availability: without investigator support
